# Using machine learning to improve our understanding of COVID-19 infection in children

**DOI:** 10.1371/journal.pone.0281666

**Published:** 2023-02-15

**Authors:** Shraddha Piparia, Andrew Defante, Kelan Tantisira, Julie Ryu

**Affiliations:** 1 Department of Pediatrics, University of California San Diego, LA Jolla, CA, United States of America; 2 Rady’s Children Hospital, San Diego, CA, United States of America; University of Wisconsin-Eau Claire, UNITED STATES

## Abstract

**Purpose:**

Children are at elevated risk for COVID-19 (SARS-CoV-2) infection due to their social behaviors. The purpose of this study was to determine if usage of radiological chest X-rays impressions can help predict whether a young adult has COVID-19 infection or not.

**Methods:**

A total of 2572 chest impressions from 721 individuals under the age of 18 years were considered for this study. An ensemble learning method, Random Forest Classifier (RFC), was used for classification of patients suffering from infection.

**Results:**

Five RFC models were implemented with incremental features and the best model achieved an F1-score of 0.79 with Area Under the ROC curve as 0.85 using all input features. Hyper parameter tuning and cross validation was performed using grid search cross validation and SHAP model was used to determine feature importance. The radiological features such as pneumonia, small airways disease, and atelectasis (confounded with catheter) were found to be highly associated with predicting the status of COVID-19 infection.

**Conclusions:**

In this sample, radiological X-ray films can predict the status of COVID-19 infection with good accuracy. The multivariate model including symptoms presented around the time of COVID-19 test yielded good prediction score.

## Introduction

SARS-CoV-2 virus, also known as beta-corona virus, has impacted global health. As of 24 April 2022, over 500 million confirmed cases and over six million deaths have been reported globally [1]. The available information on COVID-19 is constantly updated as the virus has continued infecting. The SARS-CoV-2 virus is changing over time and several variants due to mutations have been identified. According to the current literature, the typical pulmonary findings were identified as ground glass opacities and bilateral lower lobes consolidation. However, the infection is not only limited to lungs but can affect other organ disorders such as neurological side effects, kidney malfunction, etc. as well. The symptoms associated with COVID-19 infection include chills or fever, cough, shortness of breath, fatigue, etc.

Most of the early studies have focused on adults [2–4] since children were less infected with Alpha and Delta variants. However, the Omicron variant has considerably affected children, yet studies remain limited due to limited availability of data. This limitation of comprehensive data pertains to lack of testing due to mild or asymptomatic infection [5]. This study identifies the radiological findings of COVID-19 infection in children from all variants. We aim to investigate if there is a different radiographic pattern seen in COVID positive patients compared with COVID negative patients with similar symptoms since the infection often presented similarity to a typical cold or viral infections in children. Furthermore, we want to examine the effects of symptoms, history of past diseases, and demographics on COVID-19 infection by modeling them independently and in addition to radiological features. Radiological features, demographics, symptoms presented around the date of COVID test, and history of past diseases of patients younger than 18 years were collected from Epic.

## Materials and methods

### Data extraction

This study was approved by the Office of IRB Administration at University of California, San Diego 800068. The datasets for this project were compiled by querying the electronic medical record, Epic. Epic is a commercially available electronic health record software that is widely used across the United States. It is a database that health providers use to record health data in both discrete data such as vital signs, billing codes etc. and free text form such as physician notes. Free form data such as notes require natural language processing to process the data whereas discrete variables such as heartrate can be received as a single item over time. We collected information on patients who had a COVID-19 test sample collected after 3/1/2020 and had a Chest X-Ray performed within 7 days of the test sample collection date (CXR-COVID). Patients were then split into 2 cohorts; those who tested positive at least once, and those who tested negative (never returned a positive test). For patients with multiple X-Rays, the closest COVID-19 test result was kept in the dataset to avoid duplicate counts. The patients who were never tested positive were added to the negative cohort. Additionally, the X-Ray official report was collected to gather information regarding the findings of the X-Ray. To identify any pre-existing findings that may re-appear in the CXR-COVID, Chest X-Rays obtained within a year prior to the COVID test were also collected to compare the impression texts. After identifying the qualifying patients, diagnosis history (co-morbidities, problem list, and encounter diagnosis) was collected along with their responses to the patient symptom survey (Review of Systems).

### Study design and population

In this study, we used 1266 Chest X-Ray impressions (CXRis) from 721 unique COVID-19 positive patients. Ten percent of these impressions (126 in count) from 121 unique patients had a past impression available within one year of the COVID-19 test. These patients tested positive between 4/1/2020 to 1/17/2021. The remaining 1140 impressions from 600 unique patients were tested positive between 3/31/2020 to 1/20/2021 and did not have an impression from the prior year. Impressions from 1306 unique patients who tested negative for COVID-19 were used as a control. None of the COVID-19 positive patients were added to the negative cohort set. Patients that had multiple negative tests but had a CXRi along with a positive test were added to the positive group. CXRis associated with a positive test were used to investigate radiological manifestations of COVID-19 in children. All 2572 impressions were broadly divided into three categories–Normal, Stable, and New finding. All impressions were pre-processed to remove special characters, dates, etc., and converted into lower case.

**Fig 1:**
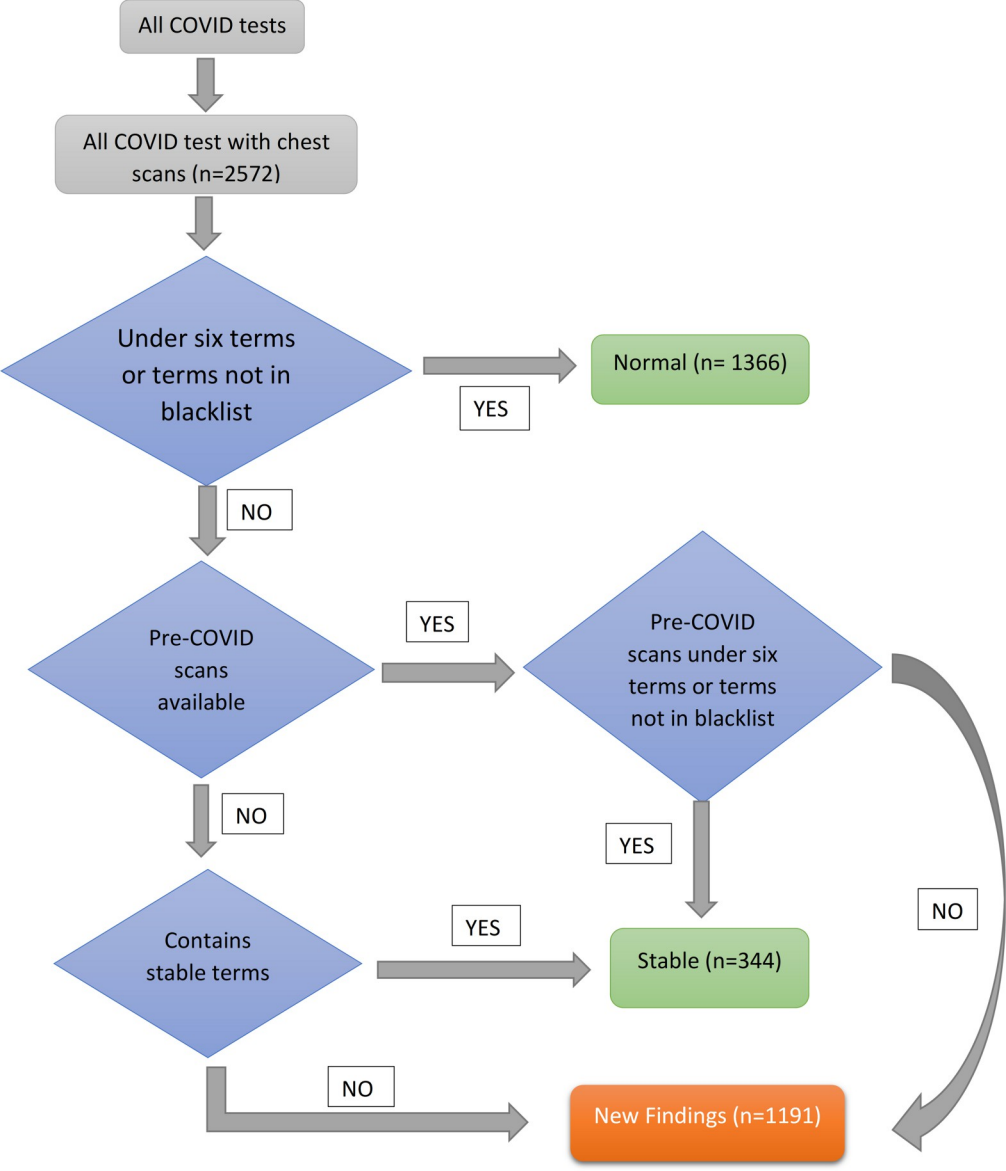
Flow chart used to divide Chest X-Ray impressions into three categories. The scans were preprocessed and then given as an input for categorization. These categories include Normal, Stable, and New findings.

Fig 1 shows the flow chart for the breakdown of pre-processed impressions into different categories. Chest X-ray scans from 2572 patients were taken along with COVID-19 tests. Each pre-processed impression was categorized as normal or abnormal based on the absence or presence of terms defined in the blacklist respectively. This is indicated in the first decision box of the flow chart which checks for two conditions. The first condition checks if the pre-processed scan has a length of less than six words since most “normal” reports consisted of 6 words or less and a second condition to check for the absence of blacklisted terms. The scan that meets either of these criterions is categorized as a normal scan. Blacklist terms included chest abnormalities such as “edema”, “nonspecific infiltrates”, “lobe pneumonia”, etc. S3 Table in S1 Data shows the terms present in different lists used for categorization of chest X-ray scans. Abnormal impressions were categorized as new findings if a past impression from the same patient was previously normal. An abnormal impression is categorized as stable if the past impression showed similar findings. In cases when a past impression is not available, it is categorized as “Stable” (or pre-existing) or “New finding” depending upon the presence and absence of terms belonging to the stable list, respectively. This list included terms such as “Stable chest”, “lungs clear”, “no significant change”, etc. S1 Table in S1 Data shows the examples of impressions for “Normal”, “Stable”, and “New finding” categories.

### Feature selection

CXRis identified as new findings were further sub-divided into specific radiographic characteristic findings. A set of 16 radiographic findings were chosen from a vocabulary of 1381 words, collective body of words from all impressions in our dataset, that were more indicative of COVID-19 infection [6, 7]. All unique terms were manually reviewed by a clinician with more than ten years of experience and findings that were linguistically different but had the same interpretation were combined. This was done to avoid redundancy since these findings will serve as features of the classifier model. It is also important to identify if these findings had any preceding and/or following negations. This was achieved by following a three-step process. In the first step, clinical terms present in the scan were identified using spaCy’s stanza en-clinical library [8]. In the next step, Chapman’s NegEx algorithm was applied to determine the presence of negation in each of these clinical terms. NegEx has a specificity of 94.5%, PPV (Positive Predictive Value) of 84.5%, and sensitivity of 77.8% [9]. In the last step, the presence of findings in these clinical terms was identified along with its negation as True or False. S2 Table in S1 Data shows an example of feature identification for an impression from a patient categorized as New finding and was COVID-19 positive.

Along with Chest X-Ray impressions, Review of System (RoS) data was obtained around the time of the COVID-19 test. The data included the presence or absence of symptoms such as fever, chills, shortness of breath, etc. Although these symptoms values were categorical, they were regrouped based on their system and assigned with a sum of their values. E.g., fever and chills belong to constitutional, shortness of breath belongs to respiratory, etc. For variant based stratification, the date of COVID test was considered. Patients tested for COVID from December 20, 2020, to April 10, 2021 were categorized as Alpha, from April 11, 2021, to November 13, 2021 were categorized as Delta, and Omicron patients were tested from November 14, 2021, to March 12, 2022.

### Random forest classifier

Random Forest Classifier (RFC) [10] is a widely used ensemble learning method for supervised classification [11]. The classifier is fast in operation and has proven phenomenally successful in several domains. Each decision tree is built by using the whole training dataset. At each node, the model chooses a feature that allows it to split the dataset into groups as diverse as possible from each other. Many uncorrelated trees are created by bagging and bootstrapping the data. This achieves a performance as close as a boosting algorithm with a classifier that is simple to train and tune. Such diverse trees operating together as a model outperform any of the individual decision trees. The model fits several decision trees and averages them to avoid over fitting as well as to improve accuracy.

There are several ways to compute and plot feature importance for an RFC–Gini importance or mean decrease accuracy, computation with permutation method, and computation with SHapley Additive exPlanations (SHAP) interpretation [12]. We chose Gini impurity and SHAP in our work. Gini impurity is a way of computing feature importance built-in the Random Forest algorithm. Each decision tree in a RFC is made up of internal nodes and leaf nodes. The internal node feature is chosen in a way that both the split nodes of the dataset have similarity within. This means that the scans categorized on one branch of main node are more similar to each other and are different than the other branch of the main node [13]. We can measure how the algorithm improves with each feature split and then average it over all trees which provides the importance score for that feature. On the other hand, SHAP is a model-agnostic interpretation. The core concept behind the SHAP interpretation is to generate partial dependence plots to compute the feature importance from Random Forest. This estimates the contribution score of each feature to the model prediction.

## Results

Pre-analysis of input data was done to determine the statistical significance of the variables examined. We used Chi-Squared test to check the dependency between categorical variables i.e., COVID-19 status and scan categories shown in Table 1, and demographic features shown in Table 2.

**Table 1:** COVID-19 positive and negative patients belonging to “New finding”, “Stable”, and “Normal” categories*.

	COVID-19 pos	COVID-19 neg	Total
**New findings**	613	554	1167
**Stable + Normal**	653	752	1405
**Total**	1266	1306	2572

* Chi-squared value for New findings vs Normal + Stable was found to be 9.54 with a p-value of 0.002 and Odds Ratio of 1.27.

**Table 2:** Distribution of COVID-19 positive and negative patients according to demographics (includes sex, race, ethnicity, and age) along with its statistical significance. These patients have CXRi, RoS, and history of past diseases which were considered for our final model *.

Demographics category	Subcategory	COVID positive (n = 1193)	COVID negative (n = 663)	Statistical Significance
**Sex**				p-value = 0.031251
	Female	593	295	OR = 1.23 (1.02, 1.49)
	Male	600	368	
**Race**				p-value = 0.001
	Asian	42	55	
	Black	101	60	
	Native Hawaiian/ Pacific Islander	5	6	
	Other (includes Hispanic/Latino/Latinx)	457	214	
	White	588	328	
**Ethnicity**				p-value < 0.00001
	Hispanic or Latino	838	331	OR = 2.37 (1.95, 2.88)
	Non-Hispanic	355	332	
**Age, years**				p-value < 0.00001
	< = 5	430	344	
	>5 < = 10	158	98	
	>10 < = 15	283	111	
	>15	322	110	
	Mean (SD)	9.24(6.66)	7.00(6.22)	
**Dichotomize age at 8.12 (mean)**	Age < = 8.12	516	416	p-value < 0.00001
	Age > 8.12	677	247	OR = 0.45 (0.37, 0.55)

*This required merging of several races together. Black includes people from Black or African American. White includes White and Middle Eastern/North African population. Asian includes Vietnamese, Filipino, Chinese, Other Asian, and Asian Indian. Native Hawaiian/Pacific Islander includes Samoan, Native Hawaiian, and Other Pacific Islander. Rest of them were counted as Other which includes Guamanian or Chamorro, Hispanic/Latino/Latinx, American Indian or Alaska Native, and Decline to Answer.

### Population characteristics and baseline radiological findings

Table 1 shows the count of patients belonging to three categories. It is evident from the table that the count of New finding is high in COVID-19 positive patients and the count of Normal is high for COVID-19 Negative patients. For further analysis, patients belonging to normal and pre-existing categories were merged into one group since they both indicated the absence of COVID-19 specific radiographic findings. Since there is a comparison between two categorical variables, a Chi-Squared test was used to determine if there was a significant difference between the proportion of “New findings” and “Normal + Stable”. This difference was observed to be significant with χ2 = 9.54, p-value = 0.002. The Odds Ratio (OR) was observed to be 1.27 with a 95% confidence interval of (1.09, 1.49).

Table 2 summarizes the sex, race, ethnicity, and age of COVID positive and negative groups. For races, we created five major categories, from 16 different races [14], as shown in the race column of Table 2. Each of these characteristics were then analyzed for statistical significance using Chi-Squared test. We found all characteristics to be statistically significant and hence added them to the model. Sex and ethnicity were also included in the model. For race, each category was represented as a binary feature. For age, we dichotomized the data and converted it into a binary feature by computing the average value of mean value of ages of COVID-19 positive and negative groups, 8.12 years. Once the datatypes were categorical, features were placed into the classification model.

### Random forest classifier modeling

**Fig 2:**
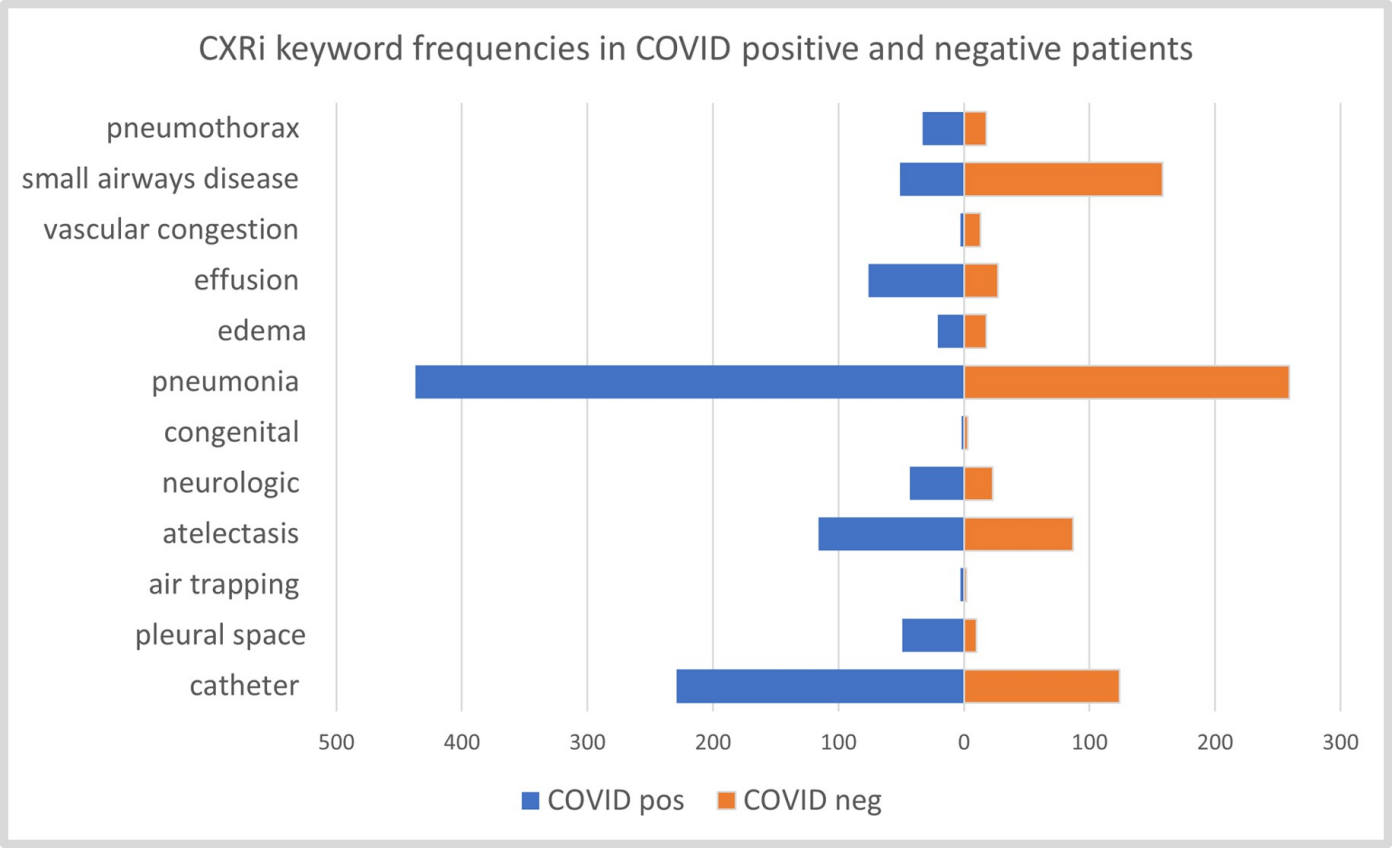
Frequencies of each feature in COVID-19 positive and negative patients.

**Fig 3:**
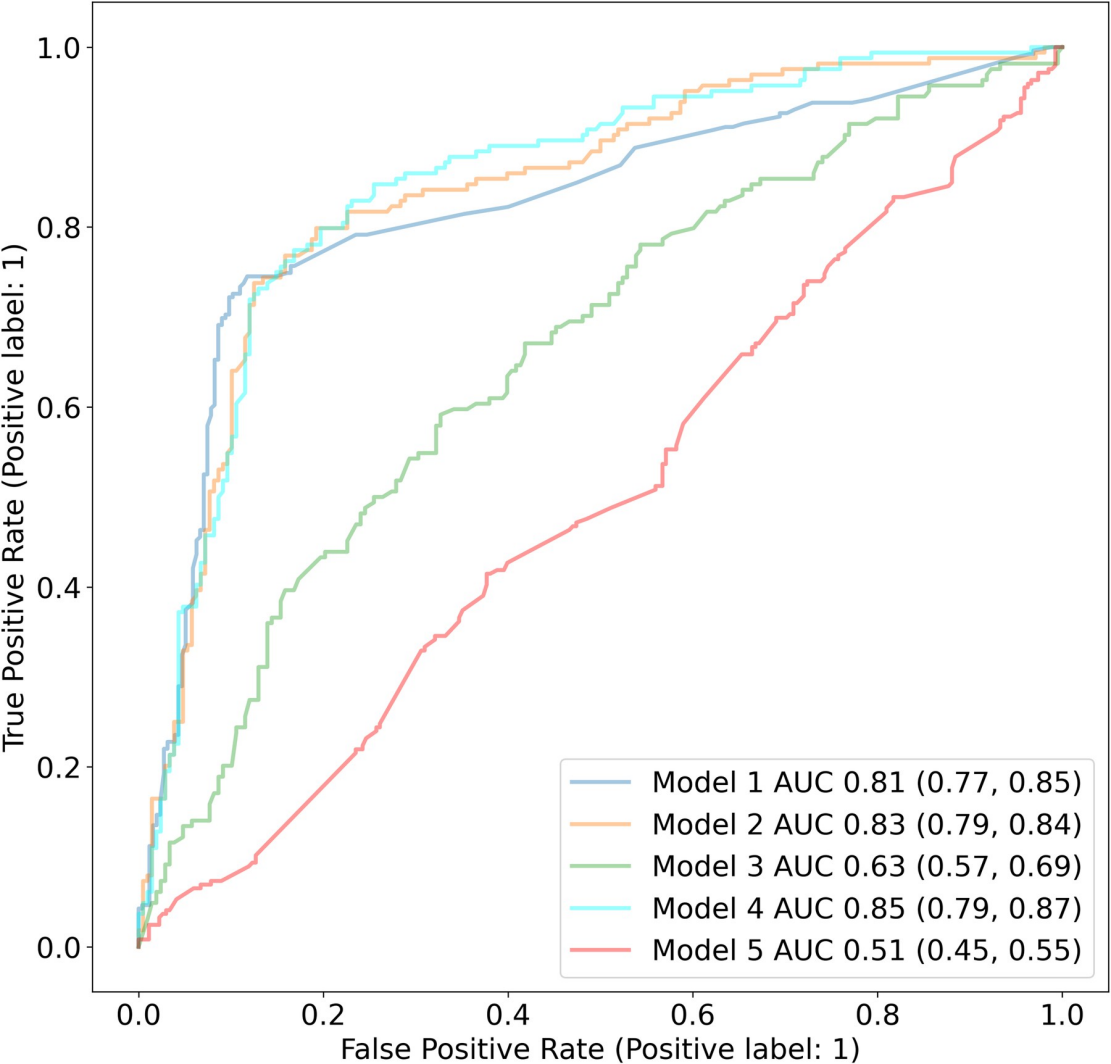
Random forest classifier performance for identifying COVID-19 infection. The ROC curves shown above identifies the ability of all ML models to classify pediatric patients with COVID-19 infection.

Fig 2 shows the cumulative frequencies of each feature in COVID-19 positive and negative patients in our dataset. The top three features in COVID-19 positive patients are pneumonia, catheter, and atelectasis. Five different RFC models were used to identify if patients have an infection or not: (1) a model based solely on the keywords from X-rays, (2) a model based on keywords from X-rays and RoS, (3) a model based on all other features i.e demographics, RoS, medical history etc. excluding the keywords from X-rays, (4) a model based on all the features i.e. X-ray keywords, RoS, demographics, and pre-existing conditions and (5) a model based solely on keywords from X-rays, where half of the data is randomly assigned a positive COVID test and half as negative COVID test (to be used as a baseline). We used GridSearchCV for hyperparameter tuning with five-fold cross validation to determine the optimal values for all models. The four parameters tuned for various values are n_estimators, max_depth, max_features, and bootstrap. The best F1 score of the models are 0.77, 0.79, 0.55, 0.79, and 0.49 respectively. We can notice that all classifiers (models 1 to 4) outperformed the baseline model 5. Model 3, which excludes the radiological features, performs a little better than the baseline indicating the importance of Xray findings. Table 3 indicates model 1 features along with their importance, odds ratio, and p-value. As observed, the top three key features for COVID prediction are pneumonia, small airways disease, and catheter. The addition of RoS, as evident by model 2 results, further strengthens the performance of classifier. Using all features in model 4 resulted in a similar F1 score of 0.79 as model 2 indicates that history of past diseases does not have a significant impact on model performance. The AUC values of all models along with their confidence intervals are shown in Fig 3.

**Table 3:** Random Forest Classifier features for model 1 in decreasing order of their importance. The feature list shown was most prominent in Chest X-Ray impressions infected with COVID-19. The table also depicts the Odds ratio along with a 95% confidence interval which was obtained using Fisher’s exact test since the sample size of these features was small.

Features	Importance	Odds ratio
**Pneumonia**	0.3518	2.83 [3.602–2.221]
**small airways disease**	0.1593	0.23 [0.32–0.162]
**Catheter**	0.1542	2.07 [2.677–1.597]
**Atelectasis**	0.1191	1.25 [1.7–0.923]
**Pneumothorax**	0.0591	1.69 [3.045–0.943]
**Effusion**	0.0509	2.76 [4.355–1.752]
**Edema**	0.0275	1.06 [2.004–0.557]
**pleural space**	0.0275	4.73 [9.425–2.37]
**Neurologic**	0.0249	1.74 [2.929–1.036]
**vascular congestion**	0.0119	0.20 [0.722–0.058]
**Congenital**	0.0109	0.6 [3.611–0.1]
**air trapping**	0.0029	1.36 [8.154–0.226]

Categories such as symptomatology (RoS, review of symptoms) around the COVID-19 test, pre-existing conditions and patient demographics were analyzed to see if there were differences between COVID-19 positive and negative groups. The demographic characteristics of patients were statistically significant (see Table 2) and were added to the model. A total of 1856 patients (1193 COVID-19 positive and 663 COVID-19 negative) were included in the CXRi model since not all patients had RoS data. To incorporate RoS to the model, the data was summarized into medical systems. A total of 12 medical systems from RoS were identified and added as features to the classifier. We noticed that only four RoS systems namely Respiratory, HENT (Head, Ears, Nose, Throat), Gastrointestinal, and Constitutional showed much greater importance than others. Hence, only the 4 most important systems were included in our feature list. These four systems included 35 individual symptoms; these 35 symptoms were then included as independent features in the model which led to an accuracy of 79.83 with an AUC of 0.83. Preexisting conditions can often affect a disease course so patient history was included in the model. The presence or absence of 21 diseases categories based on the ICD-10 codes was added to the model. In total, the classifier had 76 features after combining impressions, demographics, RoS, and medical history. The accuracy was observed to be 80.64 with an AUC of 0.85. Fig 3 shows the AUC plots of five different RF Classifiers after each increment in data. All models demonstrated a robust performance and we have described model 1, 2, and 4 in detail in the discussion section.

**Fig 4:**
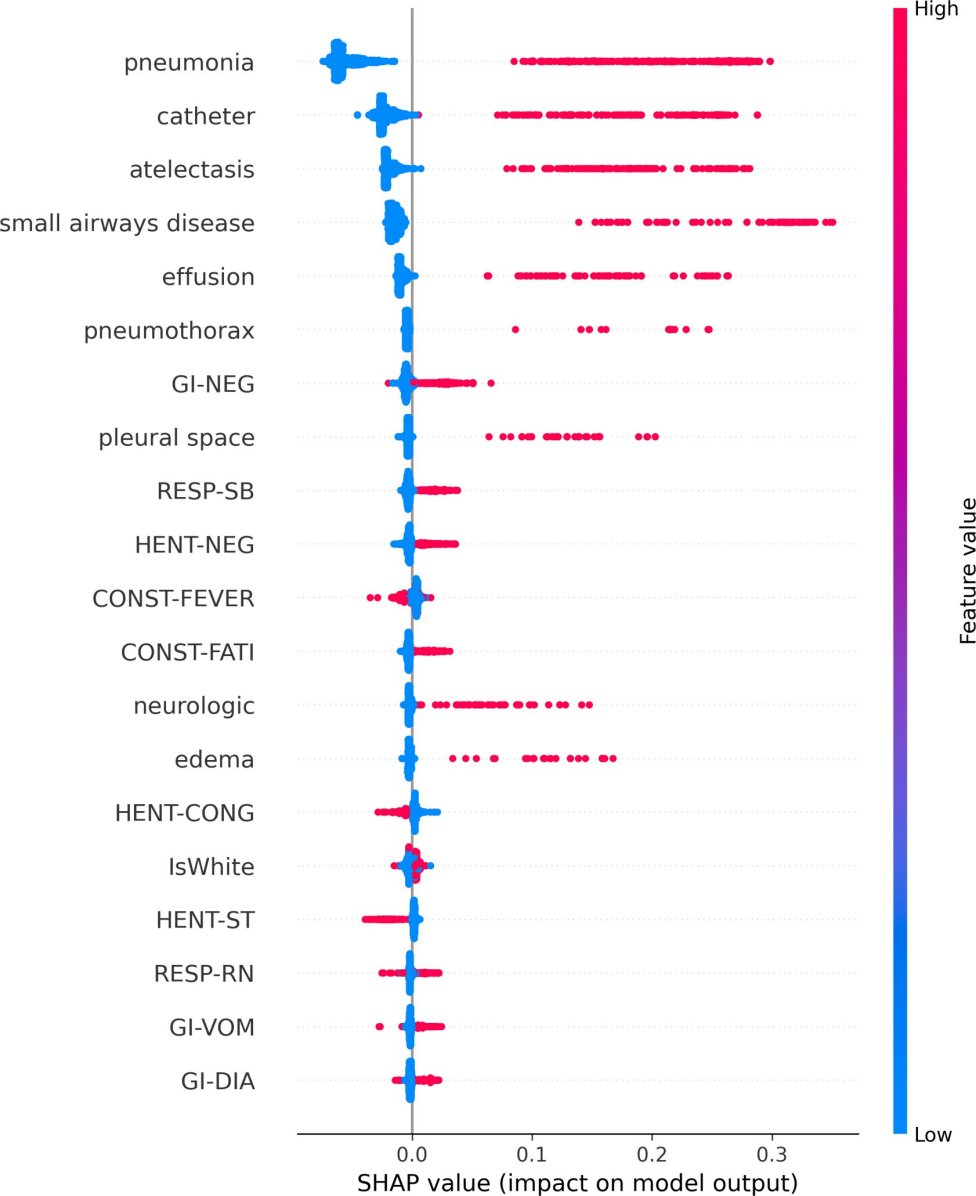
SHAP values for most important model features in model 5 shown in decreasing order of their importance along the Y-axis. Features in the upper case indicate RoS data, the lower case without an underscore represents features obtained from CXRi, and some additional demographic features. The top 20 features for the Random Forest classifier using a total of 76 features are shown using model 4. Each dot on the X-axis represents the importance value of the corresponding feature for each patient. The location of each dot indicates whether the feature is positively or negatively associated with the output. The color of each dot indicates whether the value is high (shown in red) or the value is low (indicated in blue).

Thus far, little has been known about infections of COVID-19 in children and most of the studies focus on adults. In this study, we used Random Forest Classifier to identify findings that represent COVID-19 infections. We observed that patients with symptoms related to constitutional, respiratory, and gastrointestinal systems along with the presence of pneumonia and atelectasis in CXRi contributed the most to predicting a COVID-19 infection. It is interesting to notice that the only feature that was negatively related to infection was small airways disease. To understand this better, we plotted the SHAP as shown in Fig 4. SHAP values are obtained by retraining the model by keeping most of the feature values constant and varying a feature value to determine its impact, but it is individually based so each patient will have different SHAP values.

This process is repeated by considering one feature at a time until all features are exhausted. The figure shows the top 20 most important features. We can notice from the figure that three out of the top five features are from the patient’s symptoms and the remaining two are obtained from X-Ray impressions. We also applied the RFC and SHAP model to Alpha, Delta, and Omicron patients. We included the variant subtypes because the clinical symptoms were not the same for each variant and could potentially have some difference on CXRi.

**Fig 5:**
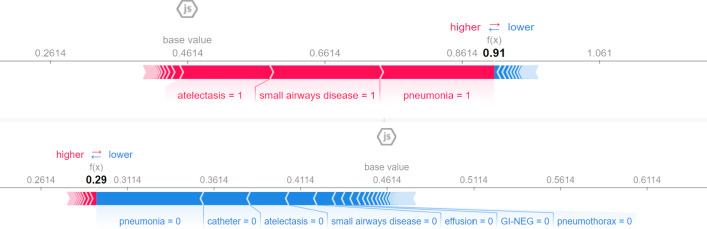
SHAP values for two patients with absence (a) and presence (b) of COVID-19 infection. The SHAP values above indicate the impact of a particular feature with a certain value in comparison to the prediction made if the feature took some baseline value. As observed in (a), the absence of pneumonia, atelectasis, and small airways disease indicates the absence of COVID-19 infection and the presence of these features in (b) indicates the presence of COVID-19 infection.

To understand the impact of these features better, we plotted the SHAP values for two individual patients as shown in Fig 5. The top and the bottom plot shows the absence and presence of COVID-19 infection, respectively.

## Discussion

Our results indicate that using radiological features is important to predict if a patient is COVID-19 positive or negative with F1 score of 0.79. CXRi findings in COVID-19 infection in children have been limited to a few studies [15–17]. Current studies have focused on adult populations and hence pediatric study remains highly understudied [18–20]. COVID-19 has had a tremendous impact on society and has been associated with significant morbidity and mortality in adult patients. Hospitalization and mortality have been associated with respiratory compromise. We investigated the impact of COVID-19 infections on children, and the respiratory system by studying the changes in chest radiographs in children who had a COVID-19 test. A simple yet effective Machine learning model was used to predict if a patient had a COVID-19 infection or not. Furthermore, two different approaches were used to analyze and validate feature importance.

The first model used only CXRi. We found that the important features used for model prediction were the features with remarkably high frequencies, and good Odds Ratio in COVID-19 positive and negative groups. These features were pneumonia, catheter, and small airways disease as shown in Table 3. Pneumonia was seen prominently in COVID-19 positive patients with a feature weight of 0.35. Small airway disease and catheter both have an importance score of 0.15 although catheter is a confounding factor. Small airway disease is commonly found in children who have asthma or asthma-like symptoms. Like the adult COVID-19 literature [21], asthma or baseline airway reactivity did not increase the probability of a COVID-19 positive test with a feature weight of 0.15. Catheter which also had a feature weight of 0.15 included the terms picc (percutaneous intravenous catheter) and line during the pre-processing step and hence may be a measure of illness which again did not alone substantially increase the probability of COVID-19 infection. The feature, atelectasis, with an importance score of 0.12 helped in predicting the COVID status of a patient. The rest of the features had a score of less than 0.05.

The second model used CXRi, symptomology report, and demographics data from all patients. This led to an accuracy of 80.65 with AUROC as 0.83. We noted that most of the top features were based on chest X-ray impression indicating that model can predict the infection with a fair accuracy by only using chest impressions. It is interesting to note that sex, ethnicity, and age played a significant role in prediction and were more predictive of the infection than symptoms. Hispanic males with age less than 8.12 had a high the chance of the infection. In addition, other studies have also reported a discordance of COVID-19 prevalence in Hispanic ethnicities [22]. It is not surprising that the top few important symptoms were gastrointestinal, fever, and congestion as literature suggests these as the common symptoms during COVID-19 infection [23]. Interestingly, sore throat was negatively associated with infection from SHAP plots. This may have resulted because the data contained a mix of several COVID-19 variants. To investigate, we stratified our data based on the variants by using the dates when COVID-19 test was administered as a marker of which variant was prevalent in our geographic area. Our data presumes 554 alpha variant patients, 309 delta variant patients, and 330 omicron variant patients by testing date for our region. The AUROC and SHAP plots are shown in S1-S3 Figs in S1 Data. We observed that the performance of the model did not degrade much for Alpha variant model. Delta and Omicron models did not yield good F1-score and AUROC probably due to the fact that these models were severely under powered. Nonetheless, the radiological features remain the main predictors of the infection and symptoms corresponded to the variant.

Lastly, a history of a patient’s pre-exiting conditions was added along with the above-mentioned features for fourth model. No specific condition was found in the top 20 important feature list indicating that the history of past diseases does not increase the likeliness of COVID-19 infection.

## Conclusions

To summarize, we implemented five Random Forest classifiers to predict the COVID-19 infection using Chest X-Ray impressions, demographics, RoS, and history of diseases in an incremental manner. Our results indicate that COVID-19 infection in children can be predicted using radiological findings. The most important radiological features observed were pneumonia, small airways diseases, and atelectasis confounded by catheter. The addition of symptoms present around testing and demographics such as sex, ethnicity, and age can help strengthen the prediction. Furthermore, the history of past diseases did not play a significant role in predicting COVID-19 infection.

## Supporting information

S1 Data (DOCX)
