# Supplementary material for: Using machine learning to improve our understanding of COVID-19 infection in children
Source: PLoS One. 2023 Feb 15;18(2):e0281666. doi: 10.1371/journal.pone.0281666 (PMC9931095; doi:10.1371/journal.pone.0281666)

# Supplementary Data

**S1 Table: Example of CXRi belonging to each category.** The Chest X-Ray impressions were divided into three mentioned categories using the flow chart depicted in Fig. I.

| CXRi category | Example |
| --- | --- |
| Normal | “lines tubes other na lungs grossly clear. no focal opacification. pleural space no evidence for pleural effusion. no evidence of pneumothorax. mediastinum cardio mediastinal contour within normal limits” |
| Stable (pre-existing finding) | 'new_scan': “preserved lung volumes. aeration improved prior. minimal retrocardiac atelectasis. no large pleural effusions pneumothoraces. unremarkable cardiac mediastinal silhouette. feeding tube terminates the stomach. central venous catheter extends atriocaval junction. stable osseous structures”  'old_scan': “broviac catheter terminates deep right atrium” |
| New finding | “increased parahilar markings suggestive mild peribronchial thickening consistent small airways disease bronchiolitis. suggestion minimal bilateral infrahilar atelectasis. no evidence of typical bacterial pneumonia air trapping” (TODO: add another example of normal past scan) |

**S2 Table: Identification of chosen categories in a sample impression after checking for negation**. Each Chest X-Ray impression was broken into sentences. All clinical terms in each sentence were identified and checked for negation. We searched for the categories present in the clinical terms and assign True or False if the finding is present or absent based on negation.

| CXRi | Categories |
| --- | --- |

| “worsening volume loss opacity, left lung  base mild mediastinal shift left. otherwise  stable streaky left perihilar right infrahilar  opacities. small residual pleural effusion  not excluded. no pneumothorax noted.  stable cardiac size. tip endotracheal tube  mid thoracic trachea. two enteric catheters  enter stomach. their distal tips scope exam.  unchanged course right upper extremity  picc extending ipsilateral neck” | {'air trapping': 0, ‘atelectasis': 0,  'catheter': 0, 'congenital': 0,  'consolidation': 0, 'covid pos': 1,  'edema': 0, 'effusion': 1,  'infiltrate': 0, 'neurologic': 0,  'new finding': 1, 'opacities': 1,  'peri-bronchial thickening': 0,  'pleural space': 0, 'pneumonia': 0,  'pneumothorax': 0, 'small airways disease': 0,  'vascular congestion': 0}. |
| --- | --- |

***S3 Table: Lists that were used for data pre-processing.***

| **Lists** | **Terms in the list** |
| --- | --- |
| **Blacklist** | edema, nonspecific infiltrates, new, large, small, infrahilar infiltrate, pulmonary infiltrate, improve, lobe infiltrate, lobe pneumonia, decrease, increase, lobe consolidation, worse, left, right, areas atelectasis, retrocardiac atelectasis, typical, stable, focal, hypoventilate, hypoexpand, hypoinflation, minimal |
| **Stable terms** | stable chest, no evidence acute cardiopulmonary disease, slightly improved aeration. otherwise unchanged, lungs clear, no significant change, no focal pneumonia, stable lung, clear lungs, within normal limits, stable exam, lungs well-expanded |
| **Normal terms** | no radiographic evidence pneumonia, lungs grossly clear. no focal opacification., no definite radiographic evidence pneumonia, normal lungs, no radiographic evidence infiltrate pneumonia, the lungs expanded. no definite focal consolidation |
| **Synonym list** | **small airways disease**: peribronchial thickening, bronchial wall thickening, small airway disease, small airways reactivity  **pneumonia**: opacity, opacities, infiltrate, consolidation  **catheter**: picc, line  **neurologic:** cardiac |

**SI Figure: SHAP values and AUROC for classifier for Alpha variant.**
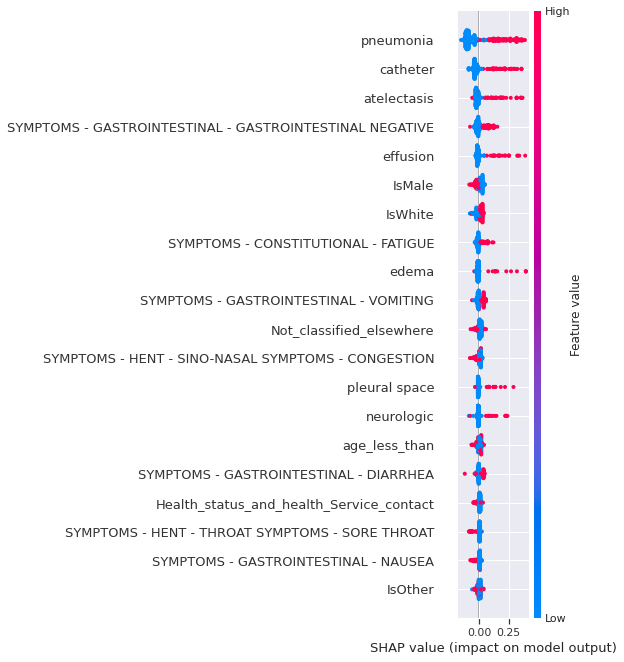


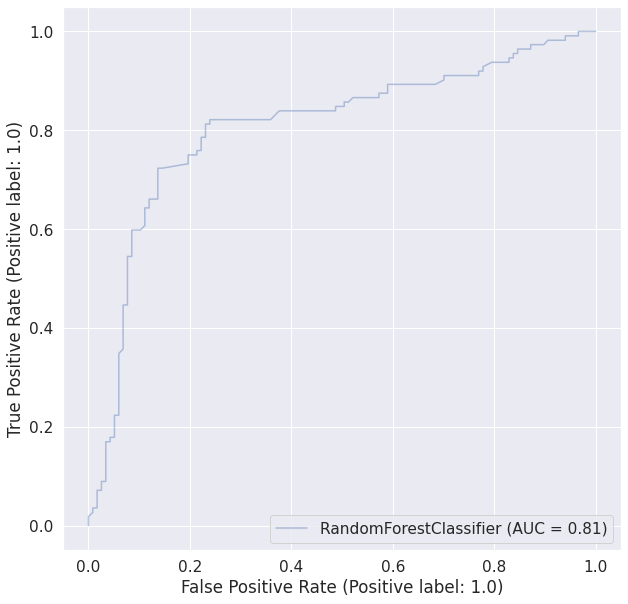


**SII
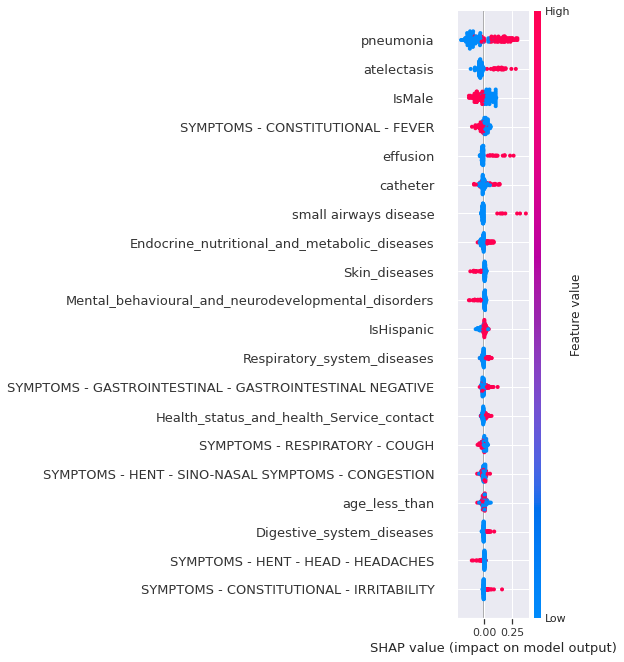
Figure: SHAP values and AUROC for classifier for Delta variant.**


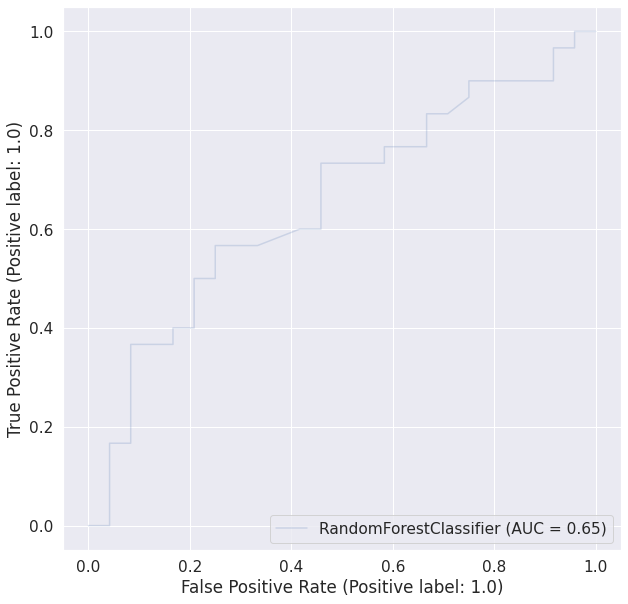


**SIII Figure: SHAP values and AUROC for classifier for Omicron variant.**


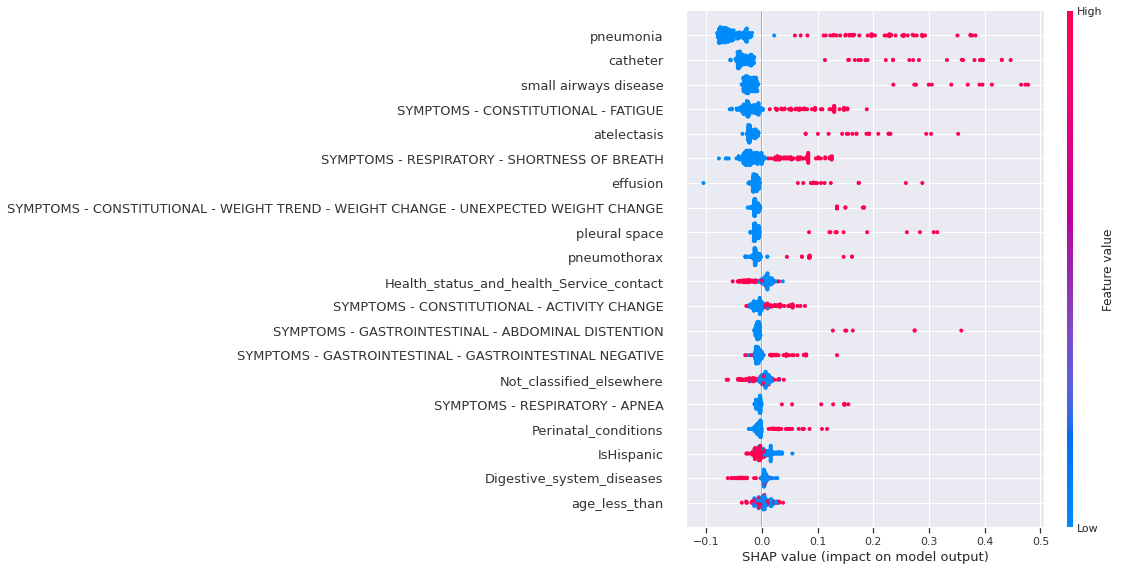

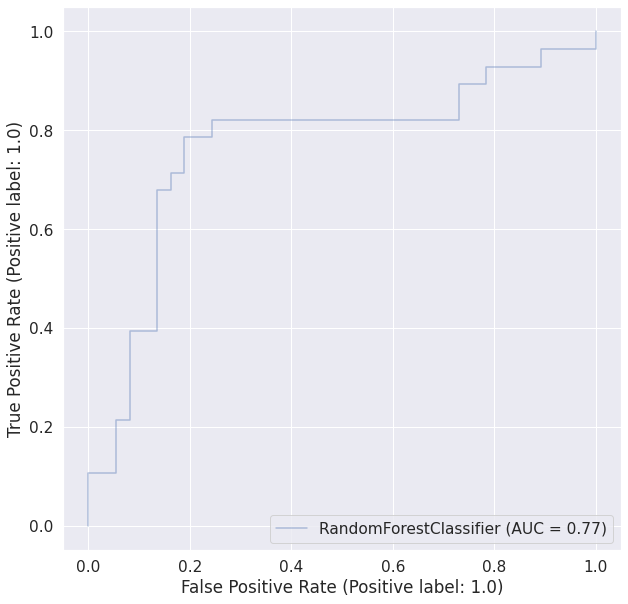

Supplement: S1 Data — (DOCX) [file pone.0281666.s001.docx]
